# Temporal Patterns in Perchlorate, Thiocyanate, and Iodide Excretion in Human Milk

**DOI:** 10.1289/ehp.9558

**Published:** 2006-11-20

**Authors:** Andrea B. Kirk, Jason V. Dyke, Clyde F. Martin, Purnendu K. Dasgupta

**Affiliations:** 1 Department of Chemistry and Biochemistry and; 2 Department of Mathematics and Statistics, Texas Tech University, Lubbock, Texas, USA

**Keywords:** breast-feeding, human milk iodide, milk, perchlorate, thiocyanate

## Abstract

**Background:**

Perchlorate and thiocyanate interfere with iodide uptake at the sodium–iodide symporter and are potential disruptors of thyroid hormone synthesis. Perchlorate is a common contaminant of water, food, and human milk. Although it is known that iodide undergoes significant diurnal variations in serum and urinary excretion, less is known about diurnal variations of milk iodide levels.

**Objectives:**

Variability in perchlorate and thiocyanate excretion in human milk has not been examined. Our objective was to determine variability of perchlorate, thiocyanate, and iodide in serially collected samples of human milk.

**Methods:**

Ten lactating women were asked to collect six milk samples on each of 3 days. As an alternative, subjects were asked to collect as many milk samples as comfortably possible over 3 days. Samples were analyzed for perchlorate, iodide, and thiocyanate by ion chromatography coupled with mass spectrometry.

**Results:**

Individual perchlorate, iodide, and thiocyanate levels varied significantly over time; there was also considerable variation among individuals. The iodide range, mean ± SD, and median for all samples (*n* = 108) were 3.1–334 μg/L, 87.9 ± 80.9 μg/L, and 55.2 μg/L, respectively. The range, mean ± SD, and median of perchlorate in all samples (*n* = 147) were 0.5–39.5 μg/L, 5.8 ± 6.2 μg/L, and 4.0 μg/L. The range, mean ± SD, and median of thiocyanate in all samples (*n* = 117) were 0.4 –228.3 μg/L, 35.6 ± 57.9 μg/L, and 5.6 μg/L. The data are not symmetrically distributed; the mean is higher than the median in all cases.

**Conclusions:**

Iodine intake may be inadequate in a significant fraction of this study population. Perchlorate and thiocyanate appear to be common in human milk. The role of these chemicals in reducing breast milk iodide is in need of further investigation.

Breast milk is widely recognized as the best source of nourishment for infants (Gartner et al. 2005). Breast-feeding also fosters an infant’s emotional and social well-being (Else-Quest et al. 2003; Winberg 2005). The American Academy of Pediatrics (Gartner et al. 2005), World Health Organization (WHO 2001), and the International Council of Nurses (ICN 2006) all recommend that infants be exclusively breast-fed for the first 6 months of life. It is important that milk be as free of detrimental agents as possible (LaKind et al. 2004); it is also important that the maternal diet provides the nutrients needed for high milk quality (Dorea 2002). This is especially true for iodine. Iodine deficiency is widely recognized as the leading and most readily preventable cause of mental impairment in children (Delange et al. 2001). Unlike adults, neonates do not have significant thyroxine stores (van den Hove et al. 1999). Exclusively breast-fed infants depend on their mother’s milk iodine for thyroid hormone (TH) synthesis and establishment of TH stores from which they can draw TH if iodine availability falls.

Thyroid hormones and therefore iodine are essential to fetal and infant neurodevelopment. Infants born to hypothyroid- or iodine-deficient women exhibit intellectual and behavioral deficits as children (Rovet and Ehrlich 2000). Such deficits may be apparent in infants as young as 3 weeks even if the degree of early deficiency was small or transient or occurred during fetal development (Kooistra et al. 2006). The Institute of Medicine (IOM 2001) recommends an iodine intake of 110 μg/day for infants 0–6 months of age, and 130 μg/day for infants 7–12 months of age. Iodine needs of pre-term infants may be twice what is needed by full-term infants (Ares et al. 2005). Breast milk–iodine content is considered sufficient when levels are 150–180 μg/L (Delange 2004). Milk samples provided by most women in our previous study (Kirk et al. 2005) fell far short of this standard. The median iodide level in human milk from 23 donors residing in 15 different states (Kirk et al. 2005) was 33.5 μg/L, and only 4 samples fell within the recommended level. We have therefore been concerned that lactating women in the United States may not be consuming sufficient iodine to meet the needs of their breast-fed infants.

Exposure to perchlorate and other iodide transport inhibitors may increase the risk of iodine deficiency among infants. The sodium–iodide symporter (NIS) is 30-fold more selective for perchlorate than for iodide and is reportedly 9–100 times as potent as thiocyanate in inhibiting iodide uptake (Dohan et al. 2003; Tonacchera et al. 2004). Perchlorate and other iodide transport inhibitors such as thiocyanate thus likely reduce transfer of iodide to breast milk at the mammary NIS. Unless major dietary changes have occurred after the birth of her child, it is also likely that a woman with perchlorate and/or thiocyanate in her milk was similarly exposed during pregnancy, potentially reducing the pool of maternal TH needed for fetal development and reducing the ability of the fetal thyroid to produce its own hormones. For a nursing infant, the production of TH would be dually impaired: first by reduction of breast-milk iodide content and then by reduced iodide uptake by the infant thyroid. A discrimination factor of 30× at both stages amounts to 3 orders of magnitude of discrimination overall. Various aspects of brain development depend precisely on when TH deficiency occurs. An infant who had insufficient TH during fetal life might suffer delay or impairment in neurologic functions that develop *in utero*. This infant may suffer other impairments if TH deficiency occurs again, or continues, after parturition. Transient or mild hypothyroidism during fetal or infant development may result in long-standing, possibly permanent functional deficits that include learning disabilities and hyperactivity (Haddow et al. 1999; Morreale de Escobar et al. 2000; Rovet 2002, 2005; Pop et al. 1999). Some find strong links between iodine deficiency and attention deficit disorders (ADD; Vermiglio et al. 2004). In the United States, an estimated 3–5% of children (approximately 2 million) have ADD (National Institute of Mental Health 2003).

A lack of data on the variability of iodide excretion limits our ability to assess milk iodine levels. Most studies of human milk iodide, including our own, have been based on single samples (Ciardelli et al. 2002; Skeaff et al. 2005), although a few have examined iodide content in samples from two (Gushurst et al. 1984; Moon and Kim 1999) or three points in time (Chierici et al. 1999). These measures may not accurately portray infant intake, especially if samples were systematically collected at times when iodide content is low. The same holds for perchlorate in milk. Although perchlorate may be common in human milk, nothing is known about the temporal variation of perchlorate levels. Finally, thiocyanate, a by-product of cyanide metabolism, is also found in human milk. We describe the variation of iodide, perchlorate, and thiocyanate levels in series of human milk samples. The implications for infant development are discussed.

## Materials and Methods

Ten lactating subjects were recruited and gave informed consent under a protocol approved by the Texas Tech University Institutional Review Board. Subjects were provided with precleaned 50-mL polypropylene tubes (Fisher Scientific, Fairlawn, NJ). Half the subjects were from the Texas Panhandle. One subject each was from Colorado, Florida, Missouri, New Mexico, and North Carolina. None reported being smokers or being vegetarian. All subjects were of European descent except for one woman residing in Texas who is of West African origin. Subjects were recruited through public notices and by word of mouth (subjects from previous studies, friends/associates of present and previous subjects). Subjects were of mid- to high socioeconomic status. Pregnant women were excluded from the study. All subjects reside in small cities or suburban environments except for one subject who resides in a small agricultural community. Samples were either expressed by pump and transferred from collection bags into provided collection tubes, or expressed manually directly into polypropylene tubes. After expression, sample tubes were placed in a supplied plastic container and stored in the donor’s home freezer until transferred to our facility, where they were maintained at –20°C. Samples were thawed before processing at 1°C. Each subject was asked to provide six samples on each of 3 days. Alternatively, subjects were asked to provide as many samples of breast milk as comfortably possible over a series of days.

Subjects provided between six and 18 samples over an average of 4.4 days (range, 2–14 days). Days were not required to be consecutive so that inconvenience to subjects could be minimized. Subjects were asked to record dates and times of sample collections and everything they ate or drank during the days that samples were collected. Food consumption was evaluated using the U.S. Department of Agriculture’s (USDA) food group tracking program “MyPyramid Tracker” (USDA 2006) and correlated with levels of perchlorate, thiocyanate, and iodide in milk. Samples were processed and analyzed by ion chromatography–mass spectrometry according to the method reported by Dyke et al. (2006).

## Results

We found considerable variability in perchlorate, iodide, and thiocyanate excretion both within and among individuals. The iodide range, mean ± SD, and median for all samples (*n* = 108) were 3.1–334 μg/L, 87.9 ± 80.9 μg/L, and 55.2 μg/L respectively. The range, mean ± SD, and median of perchlorate in all samples (*n* = 147) was 0.5–39.5 μg/L, 5.8 ± 6.2 μg/L, and 4.0 μg/L. Range, mean ± SD, and median of thiocyanate in all samples (*n* = 117) was 0.4–228.3 μg/L, 35.6 ± 57.9 μg/L, and 5.6 μg/L. The data, based on the mean and variance, are skewed to the right. Median, rather than mean, values are therefore the preferred measure for the data set as a whole. Means and variance for individuals are reported in Table 1.

On eight occasions a subject collected milk samples before consuming any food or liquid in the morning. These were matched with a sample collected after the subject’s evening meal. Student’s *t-*test demonstrated a significant increase in perchlorate levels between prebreakfast and postdinner samples (*p* < 0.03). No statistically significant difference was found for iodide or thiocyanate levels in these samples.

Milk iodine levels < 50 μg/L are considered “consistent with iodine deficiency” (Bazrafshan et al. 2005). Nearly half (46%) of all milk samples tested were below this threshold. Only 23% of the samples tested met the iodide sufficiency definition of Delange (2004). Only two of 10 donors had mean iodide levels that met the standard of iodide sufficiency when iodide concentrations were averaged. One of these subjects (C) took an iodine-containing supplement. Interestingly, samples from the other iodine-sufficient woman (E) also had the highest mean levels of perchlorate (21.4 ± 11.9 μg/L) and thiocyanate (149.6 ± 19.8 μg/L). Results for individual subjects are summarized in Table 1.

One subject reported exclusively using bottled spring water. The drinking water for two subjects came from a reverse osmosis–treated source; perchlorate levels in milk samples from these subjects were higher than the mean, with samples from one of the two volunteers (E) having the highest perchlorate content of all samples submitted (mean 21.4 μg/L, *n* = 10). Perchlorate in milk samples of the other subject drinking reverse osmosis–treated water (I) was also well above the median of 4.0 μg/L.

Perchlorate levels exceeded those of thiocyanate in 41% of the samples submitted. There was one sample each from two individuals in which perchlorate levels exceeded those of iodide. The iodide:perchlorate molar ratio ranged from 0.55 to 130 with a mean ± SD of 18.5 ± 21.9 and a median of 13.3. The thiocyanate:perchlorate molar ratio ranged from 0.016 to 267. The mean ± SD thiocyanate:perchlorate molar ratio was 18.2 ± 43.1, and the median value was 2.0. The iodide:thiocyanate molar ratio ranged from 0.21 to 251 with a mean ± SD of 33.4 ± 60.2 and a median of 3.0.

**Figure 1:**
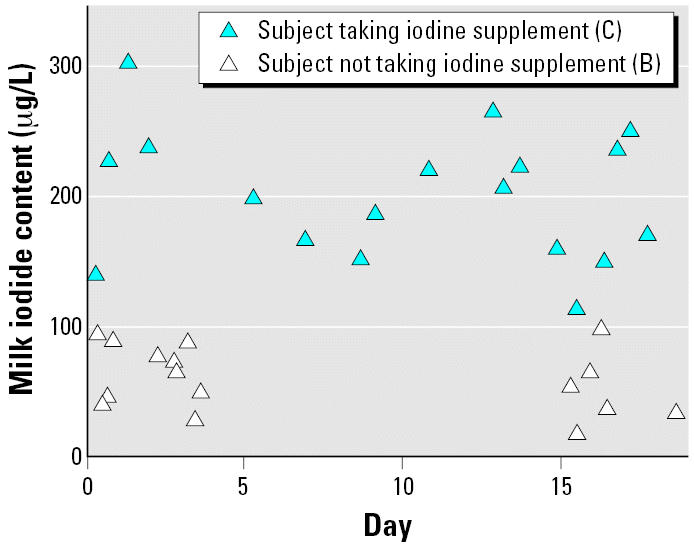
Iodide content of milk samples from two subjects (B, C). One has been taking an iodide supplement.

**Figure 2:**
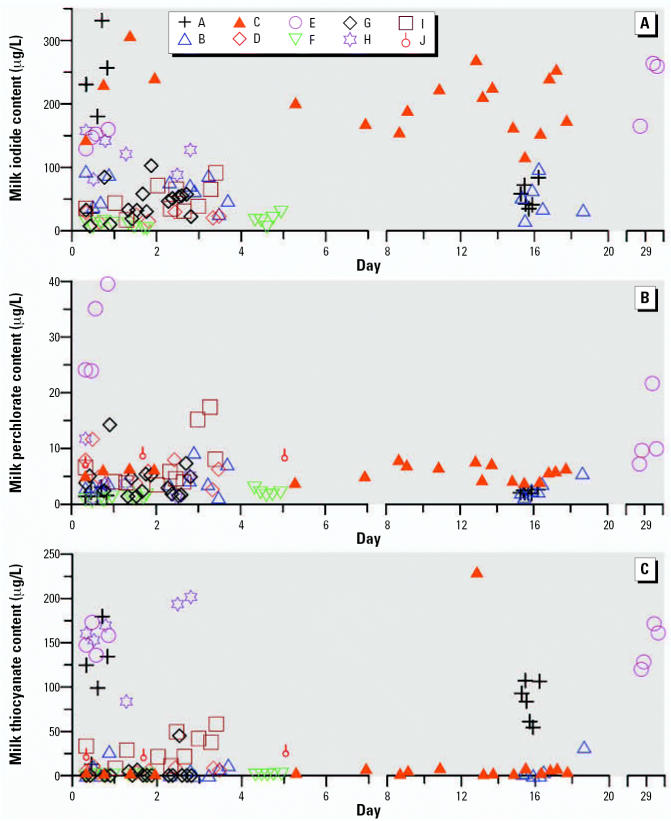
Temporal patterns for (*A*) iodide, (*B*) perchlorate, and (*C*) thiocyanate levels in human milk samples. Individual subjects are represented by symbols noted in key.

Only one subject (C) reported taking an iodine-containing nutritional supplement. Figure 1 shows iodide data for samples from this subject along with data from a subject (B) not taking an iodine-containing supplement whose median iodide values was closest to the median iodide value for the entire cohort. The difference in milk iodide content is readily noticeable. Temporal variability in iodide, perchlorate, and thiocyanate levels for all subjects are shown in Figure 2A–C.

The Centers for Disease Control and Prevention (CDC) recommend that women consume 5–9 servings of fruit and vegetables daily (CDC 2006a). Only two of our donors met this recommendation (Table 1). Perchlorate levels were positively correlated (*r*^2^ = 0.5589) with consumption of fruit and vegetables. Iodide and thiocyanate milk levels were not correlated with intake of fruit and vegetables. The unexpected lack of correlation between thiocyanate in milk and fruit/ vegetable intake may be explained by high intake of soy-based nutritional supplements along with low produce intake by one subject. When data from this and another subject with unexpectedly high thiocyanate levels are removed, the correlation increases from near zero to an *r*^2^ = 0.4758.

One donor (H) had particularly high thiocyanate levels (individual mean = 160.5 μg/L, individual median = 165.3 μg/L) when her data were compared with data from all other subjects (mean = 28.9g/L, median 5.2 μg/L) despite having the lowest level of produce intake. Although it is possible that this donor was exposed to cigarette smoke either actively or passively, she was the only subject who used soy-based nutritional beverages (mean daily intake 460 mL).

## Discussion

**Figure 3:**
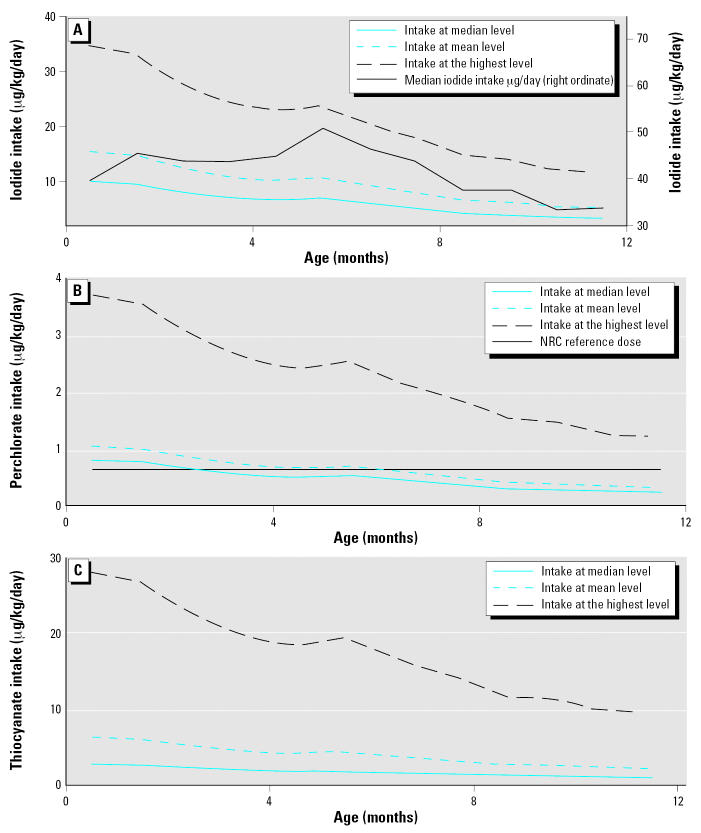
Projected iodide (*A*), perchlorate (*B*), and thiocyanate (*C*) intake of infants as a function of age corresponding to median, mean, and highest average values of each species in the sample population. See text for details.

For breast-fed infants < 12 months of age, average milk consumption is 100 mL/kg/day (Arcus-Arth et al. 2005). Three of our 10 subjects had average breast-milk perchlorate concentrations > 7 μg/L; thus for the infants of these mothers, the National Research Council (NRC) reference dose for perchlorate at 0.7 μg/kg/day (NRC 2005) will be exceeded. A more detailed analysis of breastmilk consumption as a function of age (Arcus-Arth et al. 2005), however, indicates that exposure per unit weight declines with age. Average milk intake as a function of age is available in studies by Butte et al. (1984) and Neville et al. (1988). Average infant weight as a function of age is available from the CDC (2006b); these are divided by sex—an average was used here—and the difference between the sexes is very slight. This information has been combined with our breast-milk concentration data to generate Figure 3, which shows iodide, perchlorate, and thiocyanate intake in micrograms per kilogram per day for an average-weight infant as a function of age. Each plot further shows three traces; each shows the intake of hypothetical infants consuming the mean, median, and the highest level of each species. (The mean, median, and highest values pertain to averaged data for each individual as listed in Table 1.) Figure 3A reveals that based on the median iodide content, iodine intake of our test infant population is substantially below the recommended level of 110–130 μg/day. Figure 3B indicates that if an infant of average weight, consuming an average quantity of breast milk, has a perchlorate intake corresponding to the median perchlorate content in this study, the NRC reference dose of 0.7 μg/kg/day (NRC 2005) will be exceeded for the first 2 months of his or her life. Figure 3C indicates that if perchlorate is indeed an order of magnitude or so more potent than thiocyanate in its power to inhibit iodide transport, the relative contribution of thiocyanate to iodine transport inhibition in an infant is small compared with that of perchlorate, where the infant consumes the median concentration of perchlorate and the median concentration of thiocyanate, within the limits of this study.

Single-membrane reverse-osmosis systems typically remove 80% of the perchlorate present (Foster Wheeler Environmental Corporation 1999). The fact that higher levels of perchlorate were present in milk samples from subjects drinking water treated by reverse osmosis indicates that drinking water is not necessarily the principal vector for perchlorate exposure. Moreover, one of these participants (E) used a reverse-osmosis system connected to a municipal water supply, which we have repeatedly analyzed: The perchlorate concentration in the feed water ranged from 0 to 4 μg/L, with rare excursions > 2 μg/L. Clearly, her perchlorate intake through drinking water would not account for the observed expression in breast milk. This fact—that drinking water is not generally an important vector for perchlorate exposure—is consistent with measurements of urinary perchlorate versus drinking-water perchlorate reported by Valentin-Blasini et al. (2005).

The significant difference found between prebreakfast and postdinner perchlorate levels was not surprising, given its relatively short 8-hr half-clearance time in humans (Greer et al. 2002). Thiocyanate is thought to have a half-life of 1–6 days (Junge 1985; Schulz et al. 1979), and its excretion in milk is expected to be more stable over time.

If overall intake of iodide is sufficient, it is unlikely that milk with an occasional low iodide or high perchlorate content would pose a major risk to infants. However, the data presented here, admittedly limited, indicate that the milk of many women may not supply infants with adequate iodide, while the infants are also being exposed to significant levels of perchlorate. Such infants may be at risk of altered neurologic development due to iodine deficiency with exposure to iodide-uptake inhibitors posing an additional burden. We did not measure urinary iodine content of the subjects; this, together with breast milk iodine expression, would have allowed us an estimate of the overall iodine intake of the individual. The question as to whether milk iodide is low because of iodide-uptake inhibitors such as perchlorate and thiocyanate, or whether iodide levels are low simply because maternal intake is low, cannot be answered at present without the urinary data. It may be more important to base risk assessment for perchlorate exposure on the iodide:perchlorate ratio, or the ratio of iodide to “selectivity-weighted sum of iodide uptake inhibiting agents.” If perchlorate uptake occurs 30 times more readily at the NIS *in vivo* as it appears to *in vitro* (Tonacherra et al. 2004), then an infant drinking milk with a ratio of 30:1 iodide:perchlorate may have an uptake ratio of 50:50 at its thyroid. If infants are able to use only half the iodide they receive in breast milk, potential iodine deficiency is a concern. For some individuals these ratios are relatively invariant on an order of magnitude scale, whereas wide variation is seen in others. The geometric mean of all the iodide:perchlorate molar ratio values is approximately 11. If the literature value for the *in vitro* results for iodide transport inhibition by perchlorate is applicable *in vivo*, then perchlorate may indeed be having a measurable effect on iodide transport. Similarly, the geometric mean of the iodide:thiocyanate and thiocyanate:perchlorate molar ratios are approximately 4.9 and 2.3, respectively, suggesting again that the effects of thiocyanate on iodide transport inhibition is less important than that of perchlorate in our sample population.

The real role, if any, of perchlorate in reduction of milk iodide levels is as yet unknown. Although there is evidence of inhibition from mathematical modeling (Almström 2006), this issue may be best examined through a controlled animal study; for a human study, at least simultaneous urinary data are needed to judge how intake affects expression. Our subjects had highly varied diets and varied timing of food intake; some used nutritional supplements and some did not. To further complicate correlations among analytes, the timing of milk sample collection was not uniform among donors or among days of individuals. The thyroid gland is adaptable and NIS expression increases when TH levels fall (Levy et al. 1997). NIS expression in the mammary gland is increased in response to prolactin, oxytocin, and β-estradiol (Tazebay et al. 2000), but it is not known whether mammary NIS is responsive to TSH (or some other signal) in a manner that would enable it to compensate for the presence of iodide-uptake inhibitors. Unfortunately, the degree to which the infant thyroid may be able cope with iodide-uptake inhibitors is also unknown.

Little is known about thiocyanate in human milk. To our knowledge, this is the first report of thiocyanate content of human milk in serial samples. Work was done on thiocyanate excretion in human, bovine, and rat milk during the 1960s (Funderburk and Van Middlesworth 1967). These researchers also reported that dosing rats with perchlorate reduces thiocyanate excretion in milk. Laurberg et al. (2004) reported that maternal serum thiocyanate levels are strongly correlated with low milk iodide levels. Cassava, a staple food in some African regions, contains high levels of thiocyanate and may contribute to iodine deficiency. Mean thiocyanate levels in human milk from a region of northern Zaire, where cassava consumption is high, were reported at 513 μg/L (Vanderpas et al. 1984). The highest level of thiocyanate detected in our study was less than half this amount (228.3 μg/L). The mean for our cohort was approximately 6% of the mean calculated for the northern Zaire cohort.

The present research supports earlier findings that perchlorate is common, if not ubiquitous, in the milk of U.S. women. Little information has been available on the variability of perchlorate levels in milk in individuals. This lack of information has made it difficult to assess infant exposures. An apparent insufficiency of iodine in human milk is cause for concern. U.S. infants may be at risk of iodine deficiency. Whether such risk originates in dietary insufficiency or in exposure to iodide uptake inhibitors or both is not currently known. Although removal of perchlorate or other iodide transport inhibitors from U.S. food and water supplies may be a laudable goal, this does not appear to be imminent. An effort to phase in better iodine nutrition seems warranted.

## Figures and Tables

**Table 1:** Donor food intake and mean perchlorate, iodide, and thiocyanate in breast milk.

Donor	Days	No. of Samples	Vegetable	Fruit	Grain	Milk	Meat/bean	Iodide (mean ± SD)	Perchlorate (mean ± SD)	Thiocyanate (mean ± SD)
A	2	11	78	13	114	129	67	123.6 ± 107.0	2.1 ± 0.5	96.0 ± 44.2
B	4	16	62	76	73	81	119	58.9 ± 25.6	3.8 ± 2.1	7.0 ± 9.9
C	14	18	66	26	116	54	55	201.2 ± 49.7	5.5 ± 1.3	15.4 ± 53.2
D	2	11	62	77	91	15	171	24.4 ± 7.2	7.6 ± 4.7	8.6 ± 7.0
E^a^	2	8	67	118	120	55	104	182.2 ± 55.2	21.4 ± 11.9	149.6 ± 19.8
F	3	14	72	2	166	54	55	12.0 ± 7.2	1.4 ± 0.7	0.8 ± 0.5
G	3	16	28	118	64	70	43	43.5 ± 25.6	4.2 ± 3.2	3.7 ± 11.2
H	3	6	16	35	78	32	89	119.4 ± 29.8	4.4 ± 3.9	160.5 ± 42.0
I^a^	3	10	127	85	39	33	94	49.3 ± 22.8	7.3 ± 5.0	31.2 ± 16.1
J	4	6	NA	NA	NA	NA	NA	NA	6.2 ± 2.5	15.2 ± 9.4

NA, not available; data were lost through instrument malfunction, and sufficient sample for a rerun was not available. Data on food intake are reported as percentage of recommended intake by the USDA (2006).

a Subject uses reverse osmosis water.
